
JOURNAL INFORMATION
==============================
NLM Title Abbreviation: ScientificWorldJournal
Journal ID: ScientificWorldJournal
NLM Journal ID: 101131163
Journal ID: TSWJ

The Scientific World Journal

ISSN: 2356-6140
EISSN: 1537-744X
Publisher: Wiley

ARTICLE INFORMATION
==============================
PMCID: PMC6083893
DOI: 10.1100/tsw.2001.425
Article ID: 231850
Article version: 1
Subjects: Extended Abstract

Repeated Salmon Calcitonin Injection Lowers Body Weight and Body Fat

Lutz T. A. 1
Rushing P. A. 2
Riediger T. 1
1 Institute of Veterinary Physiology, University of Zurich, Zurich, Switzerland
2 College of Medicine, Department of Psychiatry, University of Cincinnati, Cincinnati, OH, USA
Publication date: 2001
Electronic publication date: 2001 Nov 13
Volume: 1
First page: 25
Last page: 25
Copyright: Copyright © 2001 T. A. Lutz et al.
Copyright year: 2001
License: This is an open access article distributed under the Creative Commons Attribution License, which permits unrestricted use, distribution, and reproduction in any medium, provided the original work is properly cited.
License URL: https://creativecommons.org/licenses/by/3.0/

==============================
4th International Meeting on Calcitonin Gene-Related Peptide (CGRP)
*TheScientificWorld* (2001) 1(S1), 25
ISSN 1532-2246; DOI 10.1100/tsw.2001.425

### REPEATED SALMON CALCITONIN INJECTION LOWERS BODY WEIGHT AND BODY FAT

T.A. Lutz[1], P.A. Rushing[2], and T. Riediger[1]
[1]Institute of Veterinary Physiology, University of Zurich, Zurich, Switzerland; [2]College of Medicine, Department of Psychiatry, University of Cincinnati, Cincinnati, OH

Single injection of the amylin-agonist salmon calcitonin (sCT) produces a potent, long-lasting (>24 h at 5µg/kg) reduction in feeding being mediated by irreversible binding of sCT to amylin receptors. While chronic amylin administration decreases body weight gain in rats, similar experiments with sCT were not reported. We therefore tested the effect of repeated sCT injection on food intake and body weight. Once daily IP injection of sCT (1.25, 2.5µg/kg) reduced feeding in rats (40% [w/w] fat diet) by 60 [1.25 µg/kg] to 80 [2.5 µg/kg] % on day 1 and 35 to 55% on day 2. Thereafter, food intake was reduced by 20 to 30% vs. control. Over 1 week of treatment, body weight change was -33g [1.25 µg/kg] to -37g [2.5 µg/kg] vs. -5g in controls. Retroperitoneal fat was 30 to 35% lower than in controls. In a similar experiment, chow-fed rats were treated with sCT (0.3, 1.25, 2.5 µg/kg) once daily for 7 days. Food intake was reduced dose-dependently by sCT by 15 to 45% on days 1 and 2. Reduced food intake persisted (-20% vs. control) throughout the study except for the lowest dose. Over 1 week of treatment, body weight change was -10g [2.5 µg/kg], +7 g [1.25 µg/kg], +13 g [0.3 µg/kg] and +17 g [control]. Retroperitoneal fat was reduced by 25 [1.25 µg/kg] to 45 [2.5 µg/kg] % vs. control. We conclude that repeated sCT treatment potently lowers food intake in rats fed chow or a high-fat diet. Decreased food intake lead to a reduction in body weight and retroperitoneal fat mass.
